# Selection of reference genes for microRNA analysis associated to early stress response to handling and confinement in *Salmo salar*

**DOI:** 10.1038/s41598-017-01970-3

**Published:** 2017-05-11

**Authors:** Eduardo Zavala, Daniela Reyes, Robert Deerenberg, Rodrigo Vidal

**Affiliations:** 1 Department of Biology, Universidad de Santiago de Chile. Av. Libertador Bernardo O’Higgins 3363, Santiago, Chile; 2 Marine Harvest, camino el Tepual 8, Puerto Montt, Chile

## Abstract

MicroRNAs are key non-coding RNA molecules that play a relevant role in the regulation of gene expression through translational repression and/or transcript cleavage during normal development and physiological adaptation processes like stress. Quantitative reverse transcription polymerase chain reaction (RT-qPCR) has become the approach normally used to determine the levels of microRNAs. However, this approach needs the use of endogenous reference. An improper selection of endogenous references can result in confusing interpretation of data. The aim of this study was to identify and validate appropriate endogenous reference miRNA genes for normalizing RT-qPCR survey of miRNAs expression in four different tissues of Atlantic salmon, under handling and confinement stress conditions associated to early or primary stress response. Nine candidate reference normalizers, including microRNAs and nuclear genes, normally used in vertebrate microRNA expression studies were selected from literature, validated by RT-qPCR and analyzed by the algorithms geNorm and NormFinder. The results revealed that the *ssa*-*miR*-*99*-*5p* gene was the most stable overall and that *ssa*-*miR*-*99*-*5p* and *ssa*-*miR*-*23a*-*5p* genes were the best combination. Moreover, the suitability of *ssa*-*miR*-*99*-*5p* and *ssa*-*miR*-*23a*-*5p* as endogeneuos reference genes was demostrated by the expression analysis of *ssa*-*miR*-*193*-*5p* gene.

## Introduction

MicroRNAs (miRNAs) are single-stranded non-coding RNAs (ncRNAs) molecules of approximately 22 nucleotides in length, that regulate gene expression through translational repression and/or transcript cleavage in several organisms^1, 2^. miRNAs can regulate several thousands of target mRNAs, which may include up to 30% of all protein-coding genes^3^. In this context, the activity of miRNAs has been described as essential for vertebrate development and in the differentiation and/or maintenance of tissue and cell growing. In comparison with the huge amount of studies focused on the development and evaluation of miRNAs activity in plants and animal models^4, 5^, miRNA studies in fishes are relatively scarce and in particular in salmonids are scarce. However, in the last years, several studies have focused on the development and characterizaton of a great number of miRNAs in fishes and aquaculture species^6, 7^. The logic next step after these studies would be the quantitative analysis of the key miRNA of interest, which is associated to traits or particular conditions. Although currently there are several methods to detect and quantify mature microRNAs^8, 9^, the quantitative reverse transcription polymerase chain reaction (RT-qPCR), corresponds to the approach usually utilized. This approach needs the use of endogenous references genes as internal controls to normalize target gene expression, since it captures all non-biological variations^10^. The selection of reference genes to be used as internal control is not a simple approach; in fact, this selection must be conducted in a single experimental design, according to a particular moment, tissue and challenge condition. Previous studies have demonstrated that a single and universal internal control is unlikely to exist^11, 12^. The more frequently cited reference genes utilized to normalize gene expression of vertebrate miRNAs has been ribosomal RNAs, such as *18S rRNA* or small nuclear RNAs like *U6* snRNA^13–15^. However, it is very important that the referenced genes used to normalize have the same length, structure and come from the same biogenesis pathway of the interest targets genes to ensure the same results, including the same efficiency in the RNA isolation, cDNA synthesis and quantification in RT-qPCR^16^. As far as we are aware, a very limited number of studies to identify internal reference genes to miRNA have been carried out in salmonids. For example, Johansen and Andreassen^17^ have used a systematic approach to select reference-stable miRNAs in pre-smolt Atlantic salmon (*Salmo salar* L) and to validate the miRNAs selected in post-smolt individuals infected with infectious salmon anaemia virus. Likewise, Trattner and Schiller Vestergren^18^ also used Atlantic salmon pre-smolt individuals to evaluate the stability of a group of miRNAs related to lipid metabolism. However, none of these studies has evaluated the effect of stress on identification and validation of the stability of miRNAs. In farmed species like salmonids, stress conditions such as transportation, handling, confinement and crowding are considered unavoidable, because they have its origins in normal husbandry practices. Therefore, in the last decade several studies have been conducted on stress in salmonids, based principally on physiological and transcriptomic (messenger RNA) level^19–21^. A clear conclusion of these studies is that stress increases the susceptiblity to a wide variety of pathogens and, in turn, increases mortality. Thus, stress is perceived as a negative element and as a key factor determining the productivity of fish aquaculture systems.

The aim of this study was to identify and to validate systematically appropriate endogenous reference miRNA genes for normalizing the RT-qPCR survey of miRNA expression in Atlantic salmon under handling and confinement conditions associated to primary or early stress response^22^. The levels of expression of several endogenous candidate reference genes were evaluated in control and experimental stress-challenged post-smolt Atlantic salmon, including four different tissues. In consideration of our finding, we suggest the use of adequate reference genes, which, in turn, will be useful in RT-qPCR studies of miRNAs expression of Atlantic salmon under handling and confinement stress conditions.

## Results

### Efficiency and specificty of endogenous candidate reference genes

**Figure 1:**
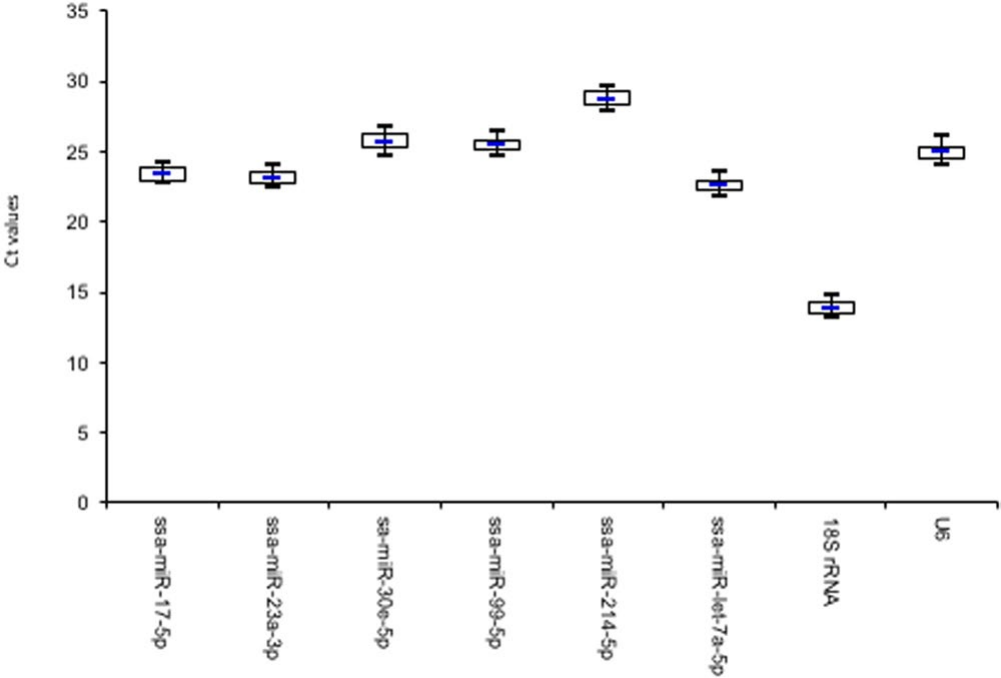
The raw threshold cycles (C_t_) data including all treatments of each reference gene in all the samples are represented in a box-and-whisker figure. Boxes represent the 25 and 75 percentiles with medians indicated. The whisker represent the highest and lowest values.

The melting curve analysis indicated, except for *ssa*-*miR*-*183*-*5p*, a single PCR product of adequate size for all the tissues, suggesting an adequate specificity of all the primers designed for these endogenous candidate reference genes. In consideration of the unspecific PCR amplification of *ssa*-*miR*-*183*-*5p*, this gene was not included in the next analysis. The results of PCR efficiency and correlation coefficients (R^2^) were very close to 100% and 1.0, respectively (Table 1). Individual threshold cycle (C_t_) values for each reference gene did not show evidence of a wide differential expression among the groups of stress treatments (step 2–4) and the control (un-stressed), with less than 1 C_t_ between the first (25th percentile) and third (75th percentile) quartiles (Fig. 1) (see Supplementary Table S1).

**Table 1:** Details of endongenous candidate reference genes.

Name*	Length (bp)	Accession number	Primer	qPCR efficiency mean (SE)	Correlation coefficient mean R^2^ (SE)
*ssa*-*miR*-*17*-*5p*	23	MIMAT0032414	F: caaagtgcttacagtgcaggtag	98.98 (0.006)	0.99 (0.002)
*ssa*-*miR*-*23a*-*3p*	21	MIMAT0032549	F: atcacattgccagggatttcc	98.97 (0.012)	1.00 (0.002)
*ssa*-*miR*-*30e*-*5p*	22	MIMAT0032611	F: tgtaaacatcctacactcagct	98.95 (0.002)	0.99 (0.005)
*ssa*-*miR*-*99*-*5p*	23	MIMAT0032718	F: aacccgtagatccgatcttgtg	98.98 (0.003)	0.99 (0.002)
*ssa*-*miR*-*183*-*5p*	22	MIMAT0032429	F: tatggcactggtagaattcact	Not included	Not included
*ssa*-*miR*-*214*-*5p*	22	MIMAT0032514	F: tgcctgtctacacttgctgtgc	99.00 (0.022)	0.99 (0.002)
*ssa*-*let*-*7a*-*5p*	22	MIMAT0032688	F: tgaggtagtaggttgtatagtt	98.99 (0.019)	0.99 (0.001)
*18S rRNA*	21	AJ427629.1	F: cgatcagataccgtcgtagtc	99.00 (0.014)	0.99 (0.002)
	18		R: cagccttgcgaccatact		
*U6*	20	BT047885	F: ttcgcgatggaagaacgcta	98.97 (0.007)	1.00 (0.001)
	20		R: aacctgctgcaagactgtgt		

*MicroRNA names in accord with^50^.

The gene *18S rRNA* showed the higher expression or abundance (mean: 13.97; SE: 0.06), The six miRNAs and the *U6* candidate genes showed smaller range of tissue expression between experimental groups, with average C_t_ values from 22.98 (SE: 0.07) to 27.27 (SE: 0.09) (Table 2).

**Table 2:** Threshold cycle (C_t_) of endogenous candidate reference genes (including all tissues and treatments). SE: standard error.

Name	C_t_ min	C_t_ max	Mean	SE
*ssa*-*miR*-*17*-*5p*	23.32	25.16	24.15	0.07
*ssa*-*miR*-*23a*-*3p*	23.25	25.07	24.03	0.07
*ssa*-*miR*-*30e*-*5p*	23.58	25.35	24.44	0.10
*ssa*-*miR*-*99*-*5p*	23.39	25.12	24.24	0.08
*ssa*-*miR*-*214*-*5p*	26.34	28.13	27.17	0.09
*ssa*-*miR*-*let*-*7a*-*5p*	22.12	23.87	22.98	0.07
*18S Rrna*	13.21	14.72	13.97	0.06
*U6*	24.74	26.42	25.53	0.11

### Plasma cortisol

**Figure 2:**
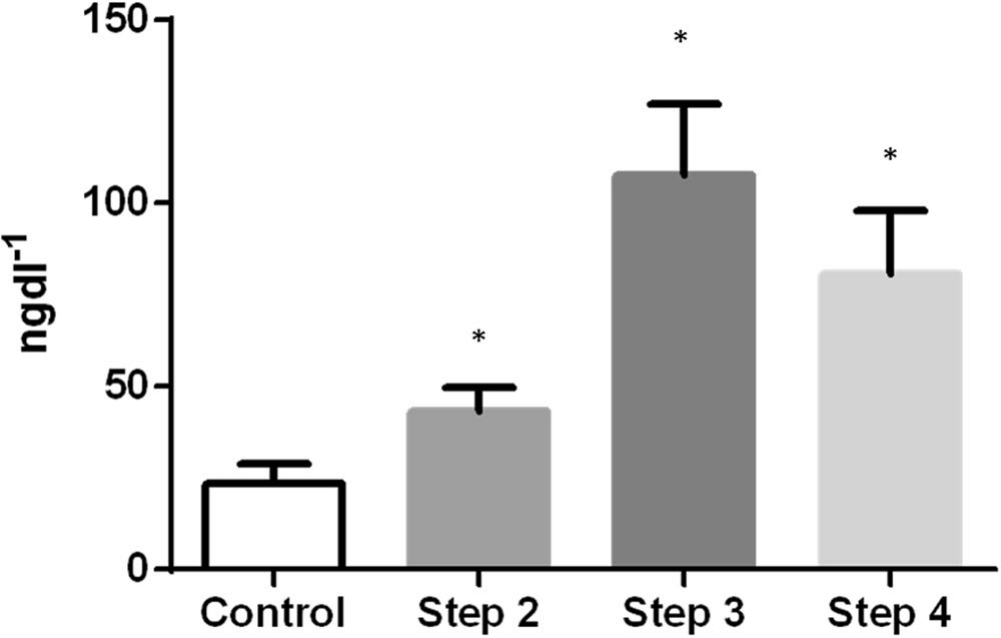
Correlation among geNorm (M value) and normFinder (stability value) results considering the pooled dataset.

Mean levels of plasma cortisol were <25 mgdl^−1^ in pre-stress conditions. A clear pattern of significantly elevated plasma cortisol was observed in post-stress conditions (Fig. 2).

### Evaluation and selection of the most stable endogenous candidate reference gene (s)

**Figure 3:**
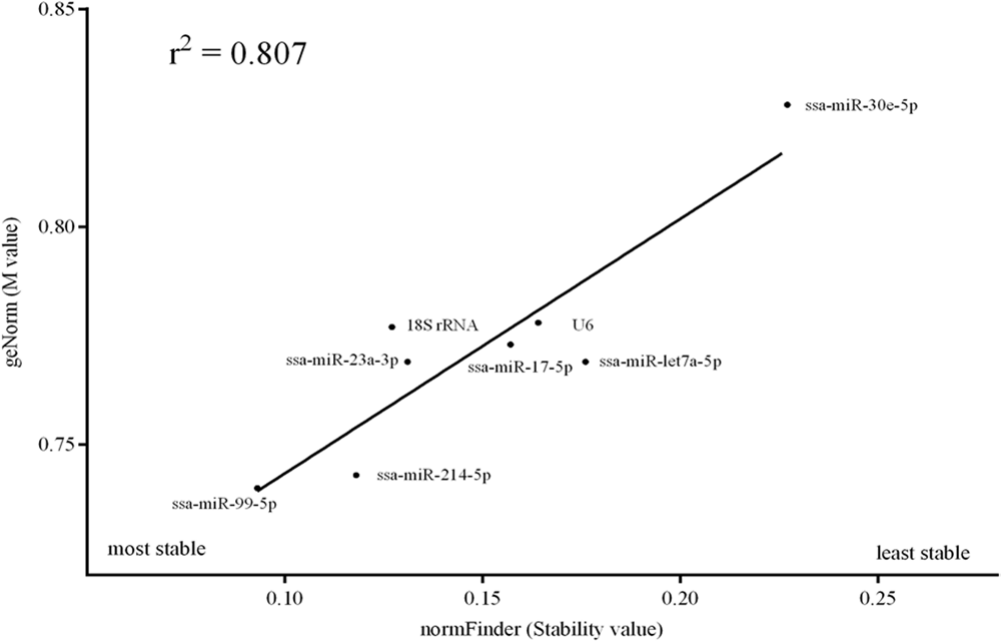
Plasma cortisol levels (±SEM) after stress experiment. *Statistical difference with respect to control (p < 0.05).

In a first stage, we evaluated the correlation between both approaches (geNorm and normFinder) considering all tissues and stress treatments, including control, pooled. The coefficient of correlation obtained between both approaches was good (r^2^ = 0.807) (Fig. 3).

Then, we utilized the two widely cited approaches geNorm and normFinder to select suitable reference gene (s). In general terms, geNorm recommends selecting genes with M value lower than 1.5 as good candidate reference gene (s), whereas NormFinder recommends choosing the candidate reference gene (s) with the lower stability value. The M values (geNorm) ranged from 0.740 to 0.828 and the stability values from 0.093 to 0.227 (normFinder). Therefore, all the genes evaluated will be potentially utilized as endogenous reference genes. However, the best stable candidate reference gene suggested by both approaches was *ssa*-*miR*-*99*-*5p* for pooled data. The individual analysis of control samples confirms *ssa*-*miR*-*99*-*5p* as the best candidate reference gene. The best combination of candidate reference genes determined by both software was *ssa*-*miR*-*99*-*5p* and *ssa*-*miR*-*23a*-*3p* (Tables 3 and 4). In the context of the selection of multiple reference genes, geNorm permits to calculate the V-value with a cut-off value suggested of ≤0.15. In the conditions of handling and confinement stress evaluated, this cut-off was obtained after V3/4 (0.134), which suggests that a minimum of three genes will be necessary for an adequate normalization. However, after V2/3, the V-value were of 0.156, a value close to the theoretical cut-off suggested. Considering the analysis by tissue in a consensus view, geNorm and normFinder ranked *ssa*-*miR*-*99*-*5p* as the most stable candidate reference gene, followed by *ssa*-*miR*-*214*-*5p*, ssa-*miR*-*17*-*5p*, *ssa*-*miR*-*23a*-*3p* and *ssa*-*miR*-*30e*-*5p* to liver, kidney, spleen and muscle, respectively (Table 5). The analysis of multiple reference genes by geNorm determined a cut-off of V 3/4 (≤0.15) for each of the tissues, nevertheless the range of values obtained for V 2/3 (0.158–0.152) were close to the theoretical value suggested.

**Table 3:** Stability and M values for all data pooled.

Name	normFinder (Stability value)	geNorm (M)
*ssa*-*miR*-*99*-*5p*	0.093	0.740
*ssa*-*miR*-*214*-*5p*	0.118	0.743
*18s rRNA*	0.127	0.777
*ssa*-*miR*-*23a*-*3p*	0.131	0.769
*ssa*-*miR*-*17*-*5p*	0.157	0.773
*U6*	0.164	0.778
*ssa*-*miR*-*let7a*-*5p*	0.176	0.769
*ssa*-*miR*-*30e*-*5p*	0.227	0.828
Best combination		
*ssa*-*miR*-*23a*-*3p and ssa*-*miR*-*99*-*5p*	0.065	
*ssa*-*miR*-*23a*-*3p and ssa*-*miR*-*99*-*5p*		0.703

**Table 4:** Stability and M values for control fish.

Name	normFinder (Stability value)	geNorm (M)
*ssa*-*miR*-*99*-*5p*	0.093	0.740
*ssa*-*miR*-*214*-*5p*	0.118	0.743
*18s rRNA*	0.127	0.777
*ssa*-*miR*-*23a*-*3p*	0.131	0.769
*ssa*-*miR*-*17*-*5p*	0.157	0.773
*U6*	0.164	0.778
*ssa*-*miR*-*let7a*-*5p*	0.176	0.769
*ssa*-*miR*-*30e*-*5p*	0.227	0.828
Best combination		
*ssa*-*miR*-*23a*-*3p and ssa*-*miR*-*99*-*5p*	0.065	
*ssa*-*miR*-*23a*-*3p and ssa*-*miR*-*99*-*5p*		0.703

**Table 5:** Stability and M values for each tissue evaluated.

Name	normFinder (Stability value)	geNorm (M value)
**Spleen**		
*ssa*-*miR*-*99*-*5p*	0.072	0.586
*ssa*-*miR*-*23a*-*3p*	0.081	0.614
*18S rRNA*	0.084	0.618
*ssa*-*miR*-*214*-*5p*	0.086	0.624
*ssa*-*miR*-*let7a*-*5p*	0.095	0.650
*ssa*-*miR*-*17*-*5p*	0.099	0.663
*ssa*-*miR*-*30e*-*5p*	0.111	0.708
*U6*	0.114	0.720
**Liver**		
*ssa*-*miR*-*99*-*5p*	0.058	0.573
*ssa*-*miR*-*214*-*5p*	0.081	0.623
*U6*	0.083	0.651
*ssa*-*miR*-*23a*-*3p*	0.091	0.668
*ssa*-*miR*-*17*-*5p*	0.097	0.679
*18S rRNA*	0.100	0.685
*ssa*-*miR*-*let7a*-*5p*	0.104	0.686
*ssa*-*miR*-*30e*-*5p*	0.111	0.716
**Muscle**		
*ssa*-*miR*-*99*-*5p*	0.066	0.647
*ssa*-*miR*-*30e*-*5p*	0.068	0.651
*ssa*-*miR*-*17*-*5p*	0.093	0.661
*ssa*-*miR*-*214*-*5p*	0.094	0.707
*U6*	0.095	0.710
*18S rRNA*	0.111	0.742
*ssa*-*miR*-*23a*-*3p*	0.127	0.786
*ssa*-*miR*-*let7a*-*5p*	0.139	0.852
**Kidney**		
*ssa*-*miR*-*99*-*5p*	0.093	0.719
*ssa*-*miR*-*17*-*5p*	0.097	0.741
*ssa*-*miR*-*let7a*-*5p*	0.104	0.752
*U6*	0.108	0.754
*ssa*-*miR*-*214*-*5p*	0.109	0.768
*18S rRNA*	0.112	0.771
*ssa*-*miR*-*23a*-*3p*	0.120	0.796
*ssa*-*miR*-*30e*-*5p*	0.125	0.808

**Figure 4:**
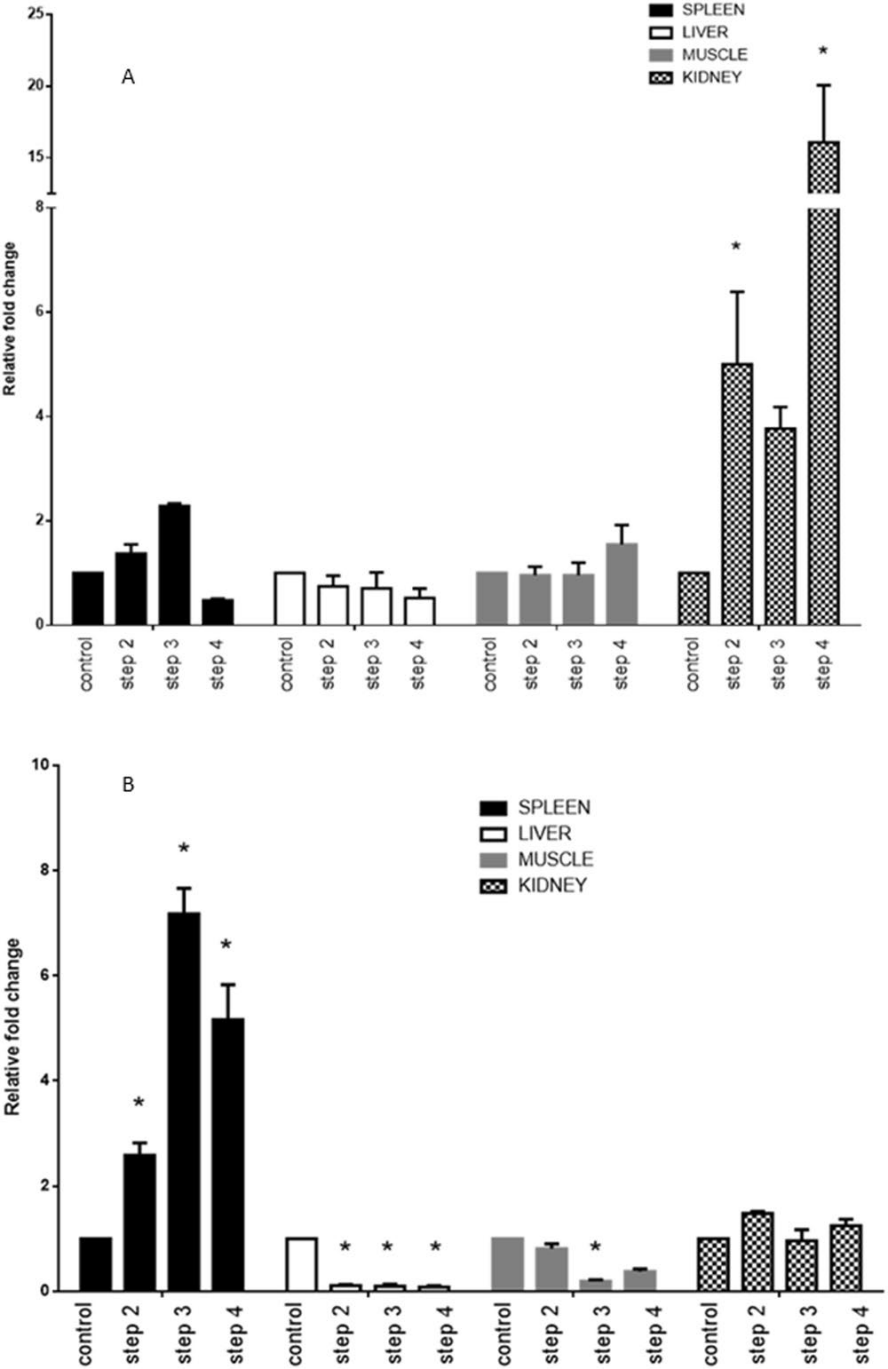
Relative expression of ssa-miR-193-5p utilizing selected candidate reference genes; *18S rRNA* for normalization (**A**) and ssa-miR-23a-3p and ssa-miR-99-5p (**B**) *p < 0.05.

To evaluate the combination of the best candidate reference genes selected, we assessed in a comparative way the expression of the selected genes versus the *18S rRNA* gene, a gene determined as least stable in the results obtained. Using the combination of *ssa*-*miR*-*99*-*5p* and *ssa*-*miR*-*23a*-*3p* as reference genes, the *ssa*-*miR*-*193*-*5p* expression in the different stress treatments for muscle and kidney tissues presented a similar general trend for remaining similar to the control level. In the case of spleen, the levels of expression were upregulated in the different stress treatment with respect to the control. A similar trend was observed in these tissues, when *ssa*-*miR*-*193*-*5p* expression was normalized with the *18S rRNA*. However, the case of the liver is noteworthy, in which the levels of expression of *ssa*-*miR*-*193*-*5p* were assigned as similar to the control in the totality of the stress treatment. This is a clear contrast with the *ssa*-*miR*-*193*-*5p* expression normalized with the two references genes selected (*ssa*-*miR*-*23a*-*3p* and *ssa*-*miR*-*99*-*5p*), since in the same stress treatments (steps 2–4) the levels of expression were downregulated with respect to the control (Fig. 4).

## Discussion

In the last years, miRNAs have received a substantial attention as stable and reproducible biomarkers associated to different biological processes and disease predictors^23–25^. Although currently different approaches exist to evaluate miRNA expression, RT-qPCR remains as the approach most regularly used. Nowadays, is widely accepted that a critical point in the design of RT-qPCR experiments correspond to the normalization strategy^26^. Despite the existence of different approaches to normalize RT-qPCR data, the endogenous reference gene corresponds to the approach more utilized^27^. Therefore, in the last years, a significant number of research has focused on the identification and validation of endogenous reference genes. Due to the capacity of miRNAs of regulating different transcript targets, even in different pathways^28^, even small changes in its expression levels may affect deeply the biological response of relevant traits. In this context, the need of counting with adequate information to miRNAs expression normalization may be more critical that to other RNA molecules. To our knowledge, this is the first report detailing identification and validation of suitable reference genes for normalization of miRNA RT-qPCR in tissues of Atlantic salmon under stress.

Initially, we selected from literature several reference genes utilized widely in miRNA RT-qPCR studies in vertebrates, and then we profiled this group of references genes in control and experimental stress conditions. This approach, to select candidate reference genes, have been widely employed in model and non-model species^29^. The aim advantage of this approach is its versatility and cost effective access to literature information. Nevertheless, recently a different approach based exclusively in RNA-sequencing data has been developed^30^. This high-throughput sequencing approach will represent a valuable opportunity to species with whole-transcriptome information available.

Our C_ts_ results showed no differences in reference genes expression level between control and experimental conditions, allowing a posterior use of geNorm and normFinder models^13^. Considering the M average expression stability of geNorm and the lowest stability values of normFinder, our results of control data indicated *ssa*-*miR*-*99*-*5p* as the candidate reference gene more stable and *ssa*-*miR*-*23a*-*3p* and *ssa*-*miR*-*99*-*5p* as the best combination. To validate the selected reference genes, the data (control and experimental) were pooled and our result confirms these reference genes as the most stable. However, Johansen and Andreassen^17^, in a recent study of miRNA validation in Atlantic salmon, suggest *ssa*-*miR*-*25*-*3p* as the most suitable reference gene and the pair *ssa*-*miR*-*25*-*3p* and *ssa*-*miR*-*455*-*p* as the best combination in organisms with infectious salmon anemia virus. These results are coherent with the ones reported previously by Dong *et al*.^28^, in the context that is necessary to select and to validate reference genes under specific experimental conditions. The selection of the optimal number of reference genes has been a critical point extensively debated in RT-qPCR researches. In theory, a single reference gene will be utilized for normalizing RT-qPCR results. However, several publications suggest results that are more reliable and use two or more reference genes^31^. In this context, the geNorm approach (V value) is normally utilized to determine the number of optimal genes. Our results suggest a minimum of three genes to normalize multi and individual tissues data of Atlantic salmon. However, the V values from geNorm represent only a general guideline and not a definitive or exact cut-off. In this context, our V-values for a minimum of two genes to normalize multi and individual tissues, are closer to the theoretical V-value of ≤0.15. Likewise, and from a practical point view, the technical complexity and cost of the assays increase with a higher number of references genes. Therefore, we considered that an adequate number of reference genes could be two. Recent miRNA expression studies in fish have utilized normalizers like *U6* and *18S rRNA* ^32–34^, probably due to: i) scarce information on endogenous reference miRNAs genes identified and validated in fishes and ii) the common use of these genes as normalizers in the literature. However, nowadays is accepted that is more appropriate to normalize gene expressions in qPCR studies with endogenous control that belongs to the same RNA category of the genes evaluated^27^. The present results are coherent with these studies and demonstrate that miRNAs show a more stable expression than other types de RNA molecules.

The analysis by tissues did not show a great variation in the ranking of the more stable reference gene (*ssa*-*miR*-*99*-*5p*), coinciding with the suggested by the pooled tissues. Nonetheless, the more stable second reference suggested varied among tissues. This is an expected result because the levels of miRNA expression could change according to a tissue specific way, affecting, in turn, its stability and M values. However, a comparative analysis of the stability and M values of pooled and separated tissues indicated a general improvement of these in separated tissues, which may be associated to the higher variability that results of combining different tissues. Therefore, in an experimental assay that considers just one tissue, the best option would be using the reference (s) gene (s) suggested to each tissue. Nonetheless, in a multi-tissue assay, they would not be the best alternative.

Normally, one approach used to validate the biological relevance of the reference candidate (s) gene (s) selected corresponds to a comparison among the expression levels of target genes normalized by the gene (s) candidate (s) and previous data reported in literature. In our case, that is not possible, because for our knowledge there is no previous data about miRNA expression in salmonid under stress conditions. Therefore, we consider to determine the practical effects of our results in a comparative way, evaluating the expression level of one miRNA gene (*ssa*-*miR*-*193*-*5p*) with *in silico* target prediction in the coding gene *heat shock protein 90*-*beta (hsp90)*, associated to the stress response in salmonid^35^. The *ssa*-*miR*-*193*-*5p* was normalized with our candidate references genes versus the *18S rRNA* gene, a gene determined as least stable in our analysis. Our results indicated a clear contrast in the levels of expression of *ssa*-*miR*-*193*-*5p*, depending of the normalizer utilized. Using our candidate reference genes selected (*ssa*-*miR*-*23a*-*3p* and *ssa*-*miR*-*99*-*5p*), the levels of expression of *ssa*-*miR*-*193*-*5p* in liver in all the experimental stressed conditions showed a clear and significant pattern of downregulation with respect to control conditions (un-stressed). This is a clear contrast with the *ssa*-*miR*-*193*-*5p* expression normalized with the *18S rRNA* gene, for which in the same stress treatments (steps 2–4) the levels of expression were similar to the control expression level. Several studies suggest an upregulation of the content of *hsp90* in salmonids with an elevated level of plasma cortisol^36, 37^. This is coherent with the present results and with the widely accepted mechanism of action of miRNA over functional coding genes^38^.

## Methods

### Tissues materials, stress challenge and cortisol

The present study is based on handling and confinement stress. For conducting this work, one pure farmed Atlantic salmon strain, Gaspe, was selected from Marine Harvest national salmon line. Two hundred post-smolt tagged individuals of Gaspe strain (unselected, mixed-sex; mean mass ± SEM: 62.18 ± 1.6 g; mean length ± SEM: 17.64 ± 0.13) were distributed evenly in 10 tanks (20 fishes by tank) and reared in standard conditions. Fish health authorities certified the experimental group as pathogen free. The fry fishes were acclimated for 14 days at 10 °C with continue commercial food feeding. The handling and confinement experimental stress was designed in 4 steps, hereafter defined as control (C) and step 2 to step 4 (S2–S4), using an adapted procedure from Carey and McCormick^39^. For control step, 10 fishes were sampled and immediately euthanized following the recommendations of Zahl *et al*.^40^. Blood samples were taken from caudal vein using a heparinized syringe and in parallel to avoid any possible temporal bias of sampling. Blood was centrifuged at 3500 × g for 7 min and plasma stored at −20 °C. The levels of plasma cortisol were measured using a commercial ELISA kit (Cortisol ELISA Kit, Neogen). Steps were compared using an analysis of variance (ANOVA) (SPSS v13 Inc., Chicago, IL) with post-hoc Tukey’s test (p < 0.05). Tissues (kidney, spleen, liver and muscle) were collected rapidly after blood sampling and preserved on DMSO-salt solution^41^. Then, the tissue were stored at −80 °C. For step 2, 10 fishes were sampled and held out of water for 30 s and then transferred to a new tank with a lower level of water by 0.5 h. The proceeding of tissue sampling and cortisol analyses were similar to the control step. For step 3, the protocol was similar to step 2 protocol, with the modification of 3 h for the phase of reduced water level. For step 4, the protocol was similar to that used in step 3, with the modification that after the reduced water level phase the fishes were returned to normal conditions and sampled after 24 h. The four steps were repeated for each tank, totalizing 400 individual samples. This experiment is part of larger research, and only 40 fishes (10 fish per control and each stress step) were chosen for this study. All the animal experiments in this study were approved and conformed to the Institutional Ethics Committee, Universidad de Santiago, guidelines.

### Endogenous candidate reference genes

Based on a literature search for genes commonly used in the normalization of microRNA expression (with a minimum of five times cited) we selected one small nuclear RNA (*U6*), one ribosomal RNA (*18S rRNA*) and seven miRNAs (*miR*-*17*-*5p*, *miR*-*23a*-*3p*, *miR*-*30e*-*5p*, *miR*-*99*-*5p*, *miR*-*214*-*5p*, *miR*-*183*-*5p* and *Let*-*7a*-*5p*) as endogenous candidate reference genes^11–13, 28–30^ (Table 1). To avoid any bias in the context of co-regulation, only genes of different miRNA families were considered, Moreover, two additional criteria were used from the RT-qPCR results: (i) expression of miRNA in all samples and tissues and (ii) low variation of expression level among experimental groups (interquartile 25^th^–75^th^ ≤1 C_t_). The conservation of the miRNA selected genes in Atlantic salmon was confirmed by Reyes *et al*.^42^.

### RNA isolation and cDNA synthesis

Total RNA were isolated using miRNeasy mini Kit, following the manufacturer’s recommendations (Qiagen, Holland), and stored at 80 °C. The RNA concentration and purity was determined by measuring the absorbance at 260 nm and 280 nm in a BioPhotometer (Eppendorf). The reversely transcribed miRNA was performed through the poly-A method, using the miRNA 1st-Strand cDNA Synthesis Kit (Agilent Technologies, Santa Clara, CA - USA), following the manufacturer’s recommendations. This method adds poly-A to the 3′ of miRNA to continue with the reverse transcription step utilizing a universal Oligo-dT adaptor. To code RNA, one μg of total RNA was reverse transcribed into cDNA using the VersoTM cDNA kit (ABgene, Surrey, UK), following manufacturer’s instructions, using random hexamers (400 ng/μL). All the products were stored at −20 °C.

### RT-qPCR of miRNAs

RT-qPCR was performed using a High-specificity miRNA RT-QPCR core kit (Agilent Technologies, Santa Clara, CA - USA) in a final volume of 25 µl. Each reaction included 2.5 μl of 10 × core PCR buffer, 2.75 μl of 50 mM MgCl_2_, 0,8 mM dNTP mix, 0.375 μl of diluted reference dye, 1.25 μl of 20 × Eva green dye, 0.125 μM universal reverse primer, 0.125 μM miRNA-specific forward primer (designed according to mature miRNA sequence), 0.5 μl of PCR enzyme blend, and 1 µL of a 1:5 dilution of cDNA. Standard curves were generated to calculate the RT-qPCR efficiency. All standard curves were generated using 5-fold serial dilutions from a pool of cDNA from all the samples (total 40 samples) and optimized in accord with the following criteria (i) linear correlation coefficient with R^2^ > 0.98 and (ii) amplification efficiencies among 95 to 110%, Reactions were incubated in a 96-well plate at 95 °C for 10 min, followed by 40 cycles of 95 °C for 10 s, 60 °C for 28 s and 72 °C for 20 s in a 7300 Real-Time PCR System (Applied Biosystems). RT-qPCR analysis of the coding nuclear gene was conducted according with the described by Cofre *et al*.^43^. All measurements were performed in triplicate. No template and minus RT controls were included. In addition, we included a minus poly-A polymerase control. For all the samples, each candidate reference gene was amplified on the same plate. Melting curve analyses, after end of each PCR, were performed to check non-specific amplification.

### Stability expression analysis

Initially, we created box plots of raw C_t_ values to evaluate, in an intuitively way, the expression stability of candidate genes. Then, the stability of all candidates genes selected was performed using two statistical approaches, geNorm^44^ and NormFinder^45^. On the one hand, NormFinder uses a mathematical model based in a variance analysis (ANOVA). This approach calculates an inter-group and intra-group variance, showing the best stable gene and the best stable pair of genes. On the other hand, the geNorm approach analyzes the gene expression stability (M) for each gene. This stability is based on pairwise variation between all candidate genes. For both statistical approaches, the C_t_ values were linearly scaled.

### Effectiveness of endongenous reference gene (s)

To evaluate the effectiveness of the reference genes chosen in an comparative way, one miRNA (*ssa*-*miR*-*193*-*5p*; CCUGUCAGUUCUGUAGGCCACU; MIMAT0032445) with target in a protein coding gene (*heat shock protein 90*-*beta*) associated to the stress response in Atlantic salmon^35^ was analyzed. Therefore, the group of candidate genes selected in this study and the *18S rRNA* gene were utilized as reference genes to normalize levels of target genes. For the prediction of the target mRNAs we used two packages RNAhybrid^46^ and miRanda^47^ to diminish the rate of false-positive results by selecting only targets sites predicted by both programs with a MFE (Minimum Free Energy) less than −28 Kcal/mol^48^. The parameters of both programs were set to seek targets with perfect complementarity of the seed region of the mature miRNA. The same group of cDNA utilized for the analysis of stability was used. The relative expression level of the miRNAs choose was obtained using the comparative C_t_ method (2^−∆∆T^)^49^. Analysis of variance (ANOVA) with post-hoc Tukey’s test (p < 0.05) (SPSS v13 Inc., Chicago, IL) were performed to evaluate statistical differences (p < 0.05).

### Ethics approval and consent to participate

All the animal experiments in this study were approved and conformed to the Institutional Ethics Committee, Universidad de Santiago, guidelines (N° 291).

## Electronic supplementary material

Table S1
