# Author Correction: Selection of reference genes for microRNA analysis associated to early stress response to handling and confinement in *Salmo salar*

**DOI:** 10.1038/s41598-018-24595-6

**Published:** 2018-04-16

**Authors:** Eduardo Zavala, Daniela Reyes, Robert Deerenberg, Rodrigo Vidal

**Affiliations:** 1 Department of Biology, Universidad de Santiago de Chile. Av. Libertador Bernardo O’Higgins, 3363 Santiago, Chile; 2 Marine Harvest, camino el Tepual 8, Puerto Montt, Chile

Correction to: *Scientific Reports* 10.1038/s41598-017-01970-3, published online 11 May 2017

In this Article, the legend of Figure 2 is incorrect:

“Correlation among geNorm (M value) and normFinder (stability value) results considering the pooled dataset.”

should read:

“Plasma cortisol levels (±SEM) after stress experiment. *Statistical difference with respect to control (p < 0.05).”

In addition, the legend of Figure 3 is incorrect:

“Plasma cortisol levels (±SEM) after stress experiment. *Statistical difference with respect to control (p < 0.05).”

should read:

“Correlation among geNorm (M value) and normFinder (stability value) results considering the pooled dataset.”

